# STEAP4 inhibits cisplatin-induced chemotherapy resistance through suppressing PI3K/AKT in hepatocellular carcinoma

**DOI:** 10.1186/s40170-023-00323-1

**Published:** 2023-12-18

**Authors:** Binhui Xie, Baiyin Zhong, Zhenxian Zhao, Jie Hu, Jianqiong Yang, Yuankang Xie, Jianhong Zhang, Jianting Long, Xuewei Yang, Heping Li

**Affiliations:** 1 Department of Hepatobiliary Surgery, the First Affiliated Hospital of Gannan Medical University, Ganzhou, 341000 People’s Republic of China; 2 Ganzhou Key Laboratory of Hepatocellular Carcinoma, the First Affiliated Hospital of Gannan Medical University, Ganzhou, 341000 People’s Republic of China; 3 Department of Hepatobiliary Surgery, The First Affiliated Hospital of Sun Yat-sen University, Guangzhou, 510080 People’s Republic of China; 4 Department of Medical Oncology, The First Affiliated Hospital of Sun Yat-sen University, Guangzhou, 510080 People’s Republic of China; 5 Department of Clinical Research Center, the First Affiliated Hospital of Gannan Medical University, Ganzhou, 341000 People’s Republic of China; 6 Department of Hepatobiliary Surgery, the Second Affiliated Hospital of Guangzhou Medical University, Guangzhou, 510000 People’s Republic of China

**Keywords:** STEPA4, Chemotherapy resistance, AKT, Hepatocellular carcinoma

## Abstract

Chemotherapy resistance is the leading cause for hepatocellular carcinoma (HCC)-induced death. Exploring resistance generation mechanism is an urgent need for HCC therapy. Here, we found STEAP4 was significantly downregulated in HCC patients with recurrence. Patients with low STEAP4 had poor outcome, suggesting STEAP4 might inhibit chemotherapy resistance. Cell viability assay, colony formation assay, apoptosis assay, soft agar growth assay, and tumor animal model showed STEAP4 inhibited cisplatin resistance. Mechanism analysis showed STEAP4 inhibited PI3K/AKT pathway through directly interacting with AKT. Double knockdown of STEP4 and AKT significantly inhibited cisplatin resistance. We also found STEAP4 expression was negatively correlated with PI3K/AKT pathway activity in clinic specimens. In summary, our findings suggested STEAP4 inhibited cisplatin resistance through suppressing PI3K/AKT pathway activity, providing a target for HCC therapy.

**Graphical Abstract:**

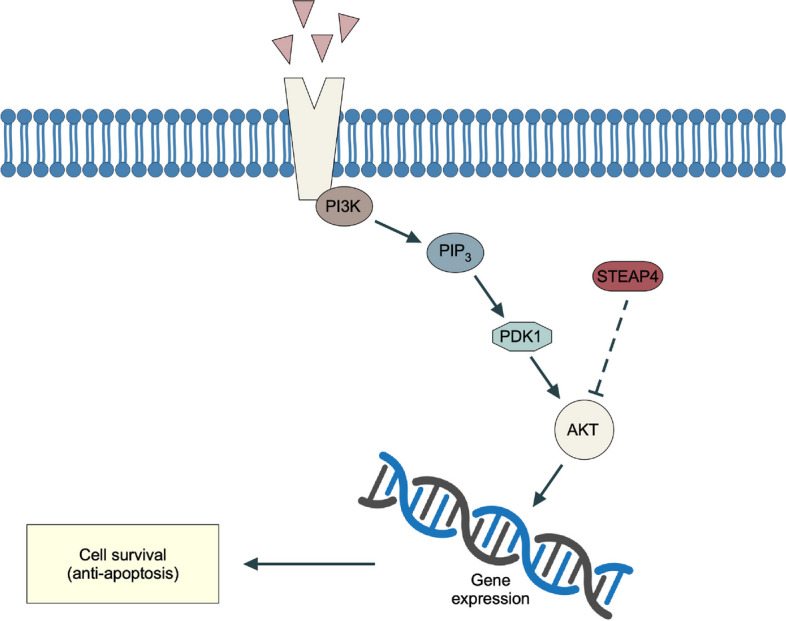

## Introduction

Hepatocellular carcinoma (HCC) is one of the most common primary malignant tumors and the third most frequent cause of cancer death worldwide [1]. Hepatitis B and C viral infections and alcohol abuse are the main risk factor for HCC initiation and development. Although these greatly improved in the last decades, its 5-year survival rate is only 15%. The resistance generation of radiotherapy and chemotherapy is the main obstacle for HCC therapy. So, it is important to understand the regulatory mechanisms of resistance generation [2].

Six-transmembrane epithelial antigen of prostate (STEAP) family proteins contain six transmembrane domains located in cell membrane. They are archaeal metal oxidoreductases and participate in tumor development. STEAP1 and STEAP2 have been reported to regulate the development of prostate cancer, breast cancer, colorectal cancer, and ovarian cancer. STEAP3 has been proposed as a candidate for prostate cancer immunotherapy and is downregulated in HCC, breast cancer, and colon cancer [3]. STEAP4 is also known as STAMP2, TNFAIP9, and TIARP, which localizes to Golgi complex, trans-Golgi network, and plasma membrane and is sensitive to androgen. It links to obesity, insulin sensitivity, metabolic homeostasis, and inflammation [4]. For example, IL-1β and TNFα regulate adipocyte metabolism. They increase STEAP4 expression in adipocytes [5, 6]. Decreased STEAP4 level is associated with visceral adipose tissue dysfunction [7]. STEAP4 level is reduced in the adipose tissues of children with obesity. It is positively correlated with high-density lipoprotein level and closely related to the blood glucose, blood lipid, blood pressure, and inflammation [8]. HBx is associated with HBV-related pathogenesis in hepatic. STEAP4 alleviates HBx-induced hepatic metabolic dysregulation and increases HBx instability [9]. High-fat diet can induce nonalcoholic fatty liver disease (NAFLD). STEPA4 improves hepatic steatosis and insulin resistance in NAFLD [10]. Drug screening find cilostazol increases MAPK pathway activity to promote STEAP4 expression, and high STEPA4 expression suppresses lipogenic factor such as LXRα and SREBP-1c expression and improves hepatic steatosis [11]. These studies suggest STEAP4 could block the progression of hepatic diseases.

The role of STEAP4 in tumor progression has been studied. It is overexpressed in prostate cancer tissues and promotes prostate cancer growth; mechanism analysis suggests that androgen increases STEAP4 expression; then STEAP4 promotes prostate cancer growth through catalyzing reduction of Fe^3+^ to Fe^2+^, and increasing NADP levels; NADP increases ROS levels, in turn promoting ATF4 expression; and ATF4 promotes prostate cancer growth [12, 13]. But its role in HCC progression has not been studied. In this study, we aimed to study the role of STEAP4 in cisplatin-induced chemotherapy resistance and found STEAP4 inhibited cisplatin-induced chemotherapy resistance through inhibiting PI3K/AKT pathway.

## Materials and methods

### Cell culture and treatment

Immortalized normal liver cell LO2 and human HCC cell lines including MSK-Hep1, SNU-475, SNU-423, HepG2, Huh7, Huh1, SNU-182, and Hep3B were purchased from the ATCC and cultured in DMEM high glucose (HyClone) supplemented with 10% fetal bovine serum (FBS), and the cells were maintained at 37 °C in 5% CO_2_ incubator. Cisplatin (DDP) was purchased from Selleck, and 30 μM was used to treat cells. Pan-Akt inhibitor GSK690693 was purchased from Selleck, and 10 nM was used to treat cells.

### Tissues samples and immunohistochemistry (IHC)

Eighteen fresh tissue specimens of HCC and three fresh tissue of non-tumor adjacent tissue, as well as 149 paraffin-embedded HCC specimens, were utilized; the detailed information was shown in Table 1. These samples were collected during surgical procedures from patients with HCC according to a protocol approved by the Institutional Review Board of the First Affiliated Hospital of Gannan Medical University. All patients provided written, informed consent for participation in the study and provision of tumor samples. IHC was performed according to our previous methods [14]. Anti-STEAP4 antibody (ab113230, Abcam) was used. The images were captured using the AxioVision Rel.4.6 computerized image analysis system (Carl Zeiss Co. Ltd., Jena, Germany).

**Table 1:** Clinicopathological characteristics of HCC patient samples

	Number of cases
**Gender**	
Male	139
Female	10
**Age (years)**	
> 45	101
≤ 45	48
**BCLC**	
0	8
1	113
2	12
3	16
**MVI**	
0	2
1	8
2	139
**Chemotherapy**	
Yes	6
No	143
**Relapse**	
Yes	106
No	43
**Pathologic differentiation**	
Well	30
Moderate	104
Poor	15
**Cirrhosis**	
Yes	39
No	110
**Fatty liver**	
Yes	8
No	141
**HBsAg**	
Yes	132
No	17
**Drinking**	
Yes	48
No	101
**Smoking**	
Yes	68
No	81
**Survival**	
Yes	108
No	41
**STEAP4**	
High level	68
Low level	81

### Vectors, lentiviral infection and transfection

Human STEAP4 cDNA was subcloned into the pSin-EF1α-puro lentiviral vector to generate pSin-EF1α-STEAP4 vector (indicated as STEAP4); the empty vector was used as the negative control (indicated as vector). Two short hairpin RNAs (shRNAs) oligonucleotide sequences against STEAP4 were cloned into the PLKO.1 lentiviral vector to generate PLKO.1-STEAP4 shRNAs (indicated as shRNA#1 and shRNA#2, respectively). The sequences of shRNAs were as follows: shRNA#1, 5′ GCAGGTGTTTGTGTGTGGAAA3′ and shRNA#2, 5′ CCAAGCAAAGAGTGATGGATA3′. The scramble shRNA sequence was cloned PLKO.1 vector and used as the negative control (indicated as Scramble). These vectors were cotransfected with pM2.G and psPAX2 into 293T using Exfect Transfection Reagent (Vazyme, Nanjing, China). The lentiviral supernatants were collected 48 h after transfection and filtered through a 0.45-μm filter. Supernatants plus polybrene (Sigma) were infected with growing HCC cells; after 12 h, the supernatants were replaced by fresh medium. Puromycin (Sigma) was used to screen stably cell lines.

### qRT-PCR

Total RNA was extracted using RNA isolater Total RNA Extraction Reagent (Vazyme), and reversely transcribed into cDNA using HiScript II first-strand cDNA synthesis kit with gDNA wiper (Vazyme). Relative gene expression levels were examined using AceQ qPCR SYBR Green Master Mix (Vazyme) on a CFX96 Touch Real-Time PCR Detection system (Bio-Rad). GAPDH was used as the internal control.

### Western blot

Total proteins were extracted using RIPA buffer (50-mM Tris (pH 7.4), 1-mM EDTA, 150-mM NaCl, 1% NP-40, 0.5% sodium deoxycholate) supplemental with protease inhibitors (Roche). Antibodies against STEAP4 (ab113230, Abcam), p-AKT (ab105731, Abcam), AKT (#9272, CST), and GAPDH (G8795, Sigma) were used. GAPDH was used as the loading control.

### Cell viability assay

MTT assay and terminal deoxynucleotidyl transferase nick end labeling (TUNEL) assay were performed using our previous methods [14].

### Colony formation assay

Cells were seeded in 6-well plates and continued to culture in fresh medium for 14 days; DDP was used to treat cells. Crystal violet was used to stain colonies; for quantification, colonies formed by more than 50 cells were scored.

### Soft agar growth assay

One-hundred cells were suspended in 0.4% agar in DMEM high glucose medium containing 10% FBS and plated on 1% agar in 6-well plates. The plates were incubated for 2 weeks. Colonies who contain over 50 cells per colony were counted to determine the efficacy.

### In vivo xenograft assay

All animal experiments were performed under the protocols approved by the Institutional Animal Care and Use Committee of the First Affiliated Hospital of Gannan Medical University. Six-week-old BALB/c-nu mice were purchased from the Experimental Animal Center of the Guangzhou University of Chinese Medicine. In total, 5 × 10^6^ Hep3B with STEAP4 overexpression or knockdown were orthotopically injected into the liver parenchyma of mice (*n* = 6) to observe the tumor growth, and mice were intraperitoneally treated with DDP (5 mg/kg) twice per week for 3 weeks. The mice were continued to feed for 40 days and then were euthanized, and tumors were excised.

### Statistical analysis

SPSS 19.0 was used to perform all statistical analyses. All data from at least three independent experiments are presented as the mean ± SD. Comparisons between different groups were analyzed using Student’s *t*-test. Survival curves were derived from Kaplan-Meier estimates, and multivariate Cox-regression analysis was used to determine the prognostic value of STEAP4 levels and other clinicopathologic characteristics. RNA-seq data from the TCGA HCC data set portal were used for analyzing STEAP4 expression, and Salmon and DESeq2 were used to analyze STEAP4 expression in HCC samples and normal liver samples. Gene set enrichment analysis (GSEA) was performed using GSEA 2.0.9 software http://software.broadinstitute.org/gsea/index.jsp. *P* < 0.05 was considered to be statistically significant.

## Results

### STEAP4 is negatively associated with HCC prognosis

**Fig. 1:**
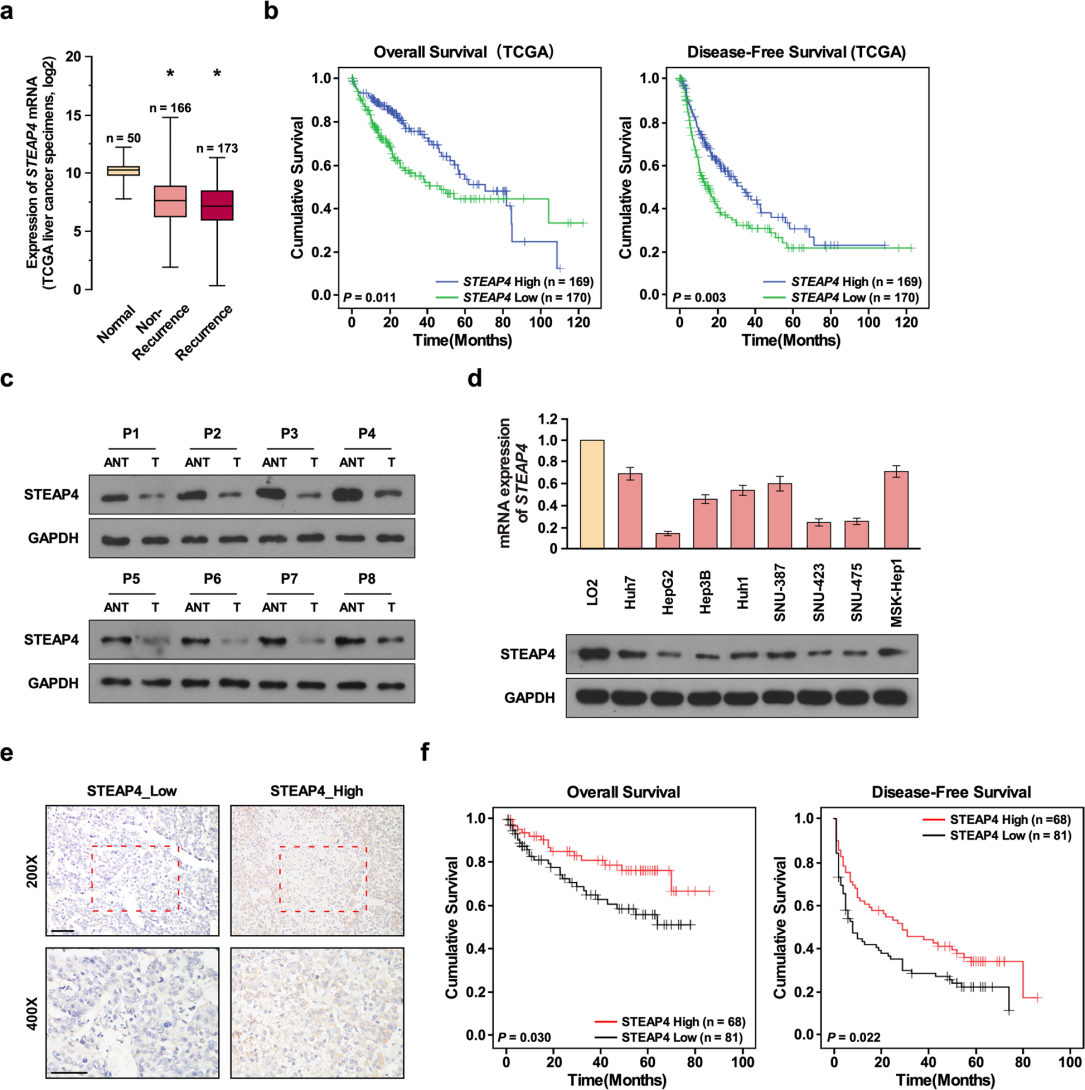
High STEAP4 expression correlates with good prognosis in patients with HCC. **a** STEPA4 mRNA levels in HCC TCGA dataset. **b** Overall and disease-free survival of patients in TCGA HCC dataset with low versus high STEAP4 mRNA levels. **c** Western blot analysis of STEPA4 expression in HCC tissues (T) and adjacent normal liver tissues (ANT). **d** q-PCR analysis of STEAP4 expression in normal liver cell line and HCC cell lines. **e** Representative images of IHC staining of 149 primary HCC samples with high STEAP4 expression and low STEAP4 expression. Top, 200× magnification; bottom, 400× magnification. **f** Overall and disease-free survival of HCC patients with high versus low STAEPA4 expression. Bars represent the mean ± SD of three independent experiments; *P < 0.05

To determine the role of STEAP4 in HCC progression, we first examined whether STEAP4 was associated with HCC prognosis. Through analyzing TCGA dataset, we found STEAP4 was significantly downregulated in HCC tissues compared to normal liver tissues, especially in HCC tissues with recurrence (Fig. 1a). Overall and disease-free Kaplan-Meier analysis showed that patients with low STEAP4 expression had shorter survival time than patients with high STEAP4 expression (Fig. 1b), suggesting STEAP4 might be negatively associated with HCC progression. To confirm above results, we determined STEAP4 expression in HCC tissues and adjacent normal liver tissues and in HCC cells and normal liver cell. Western blot and Q-PCR analysis showed STEAP4 was low in HCC tissues and cells (Fig. 1c and d). We also determined STEAP4 expression in 149 HCC tissues using IHC (Fig. 1e). Overall and disease-free Kaplan-Meier analysis showed patients with low STEAP4 had shorter survival time than patients with high STEAP4 expression (Fig. 1f). These results suggested that STEAP4 was low expression in HCC tissues and was negatively associated with patients’ survival.

### STEAP4 inhibits chemotherapy resistance

**Fig. 2:**
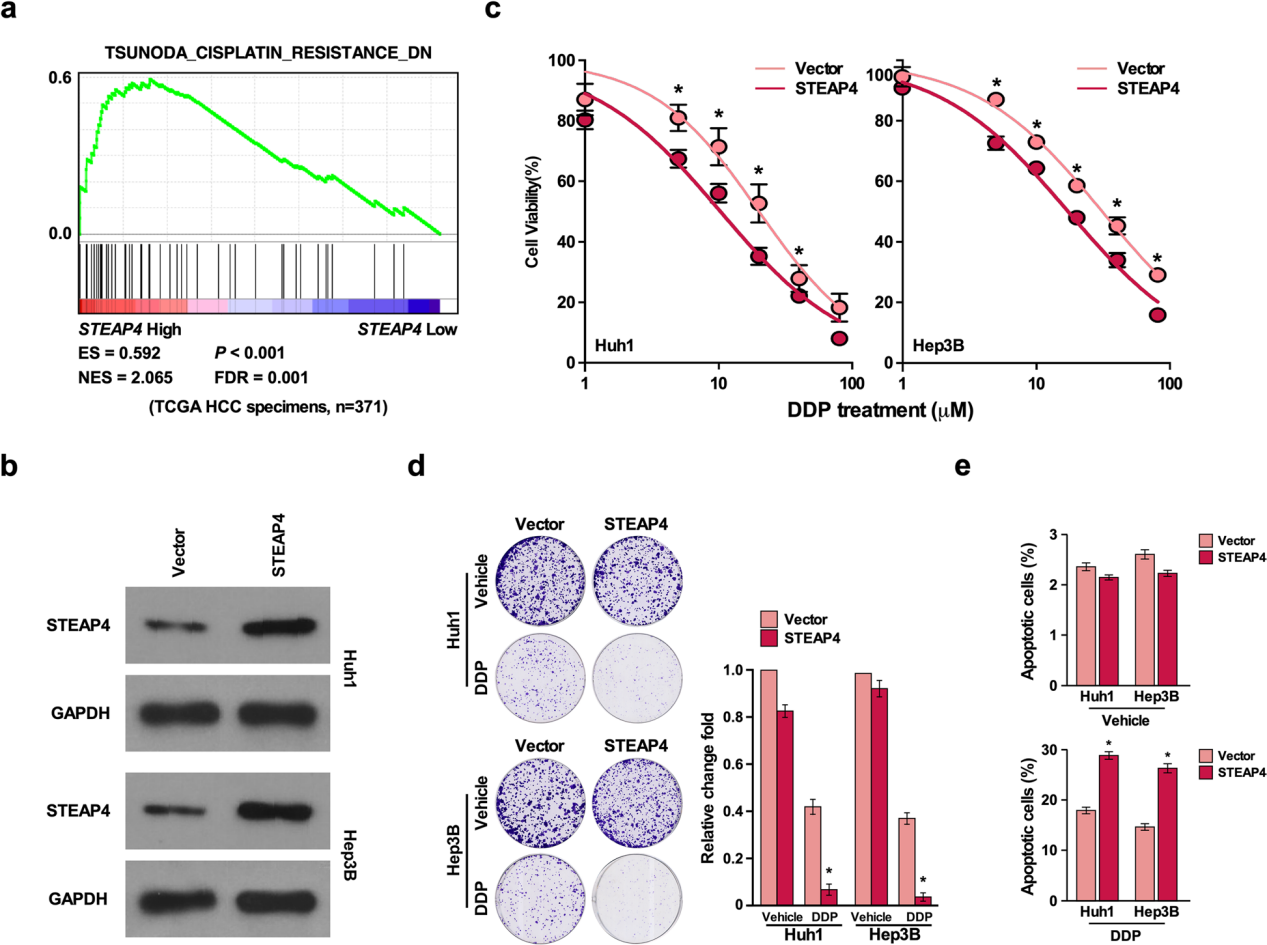
STEAP4 overexpression inhibits DDP-induced chemotherapy resistance *in vitro*. **a** GSEA indicating significant correlation between STEAP4 expression and DDP-induced chemotherapy resistance generation. **b** Western blot analysis of STEAP4 expression in the indicated STEAP4-infected cells. GAPDH was used as the loading control. **c** Cell viability analysis for the effect of STEAP4 overexpression in DDP-induced cell proliferation. **d** Colony formation analysis for the effect of STEAP4 overexpression in DDP-induced cell proliferation. **e** Annexin/PI apoptosis analysis for the effect of STEAP4 overexpression in DDP-induced cell apoptosis. Bars represent the mean ± SD of three independent experiments; *P < 0.05

**Fig. 3:**
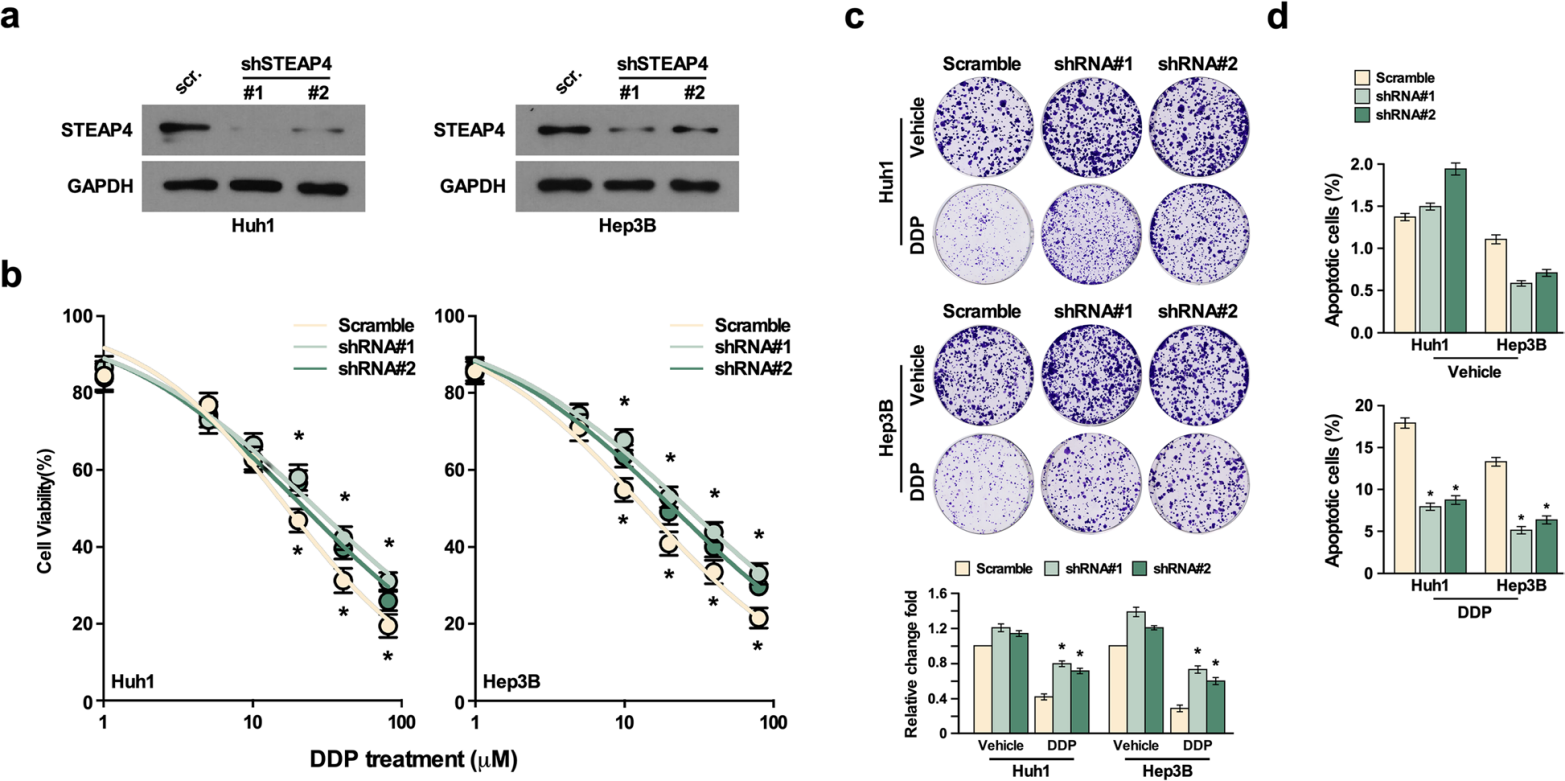
STEAP4 knockdown promotes DDP-induced chemotherapy resistance *in vitro*. **a** Western blot analysis of STEAP4 expression in the indicated STEAP4 shRNA-infected cells. GAPDH was used as the loading control. **b** Cell viability analysis for the effect of STEAP4 knockdown in DDP-induced cell proliferation. **c** Colony formation analysis for the effect of STEAP4 knockdown in DDP induced cell proliferation. **d**. Annexin/PI apoptosis analysis for the effect of STEAP4 knockdown in DDP-induced cell apoptosis. Bars represent the mean ± SD of three independent experiments; *P < 0.05

To determine whether STEAP4 regulated chemotherapy resistance of HCC, GSEA analysis showed that low STEAP4 expression was positively correlated with cisplatin resistance (Fig. 2a), suggesting STEAP4 might regulate chemotherapy resistance. To confirm this conference, we overexpressed and knockdown STEAP4 in two HCC cell lines Huh1and Hep3B, respectively. Western blot was used to confirm the effect of overexpression and knockdown (Figs. 2b and 3a). Cell survival assay showed that STEAP4 overexpression increased cell death after treating with DDP compared to vector control, while STEAP4 knockdown increased cell survival after treating with DDP compared to scramble control (Figs. 2c and 3b). Colony formation assay showed DDP treatment inhibited HCC cell proliferation compared to vehicle group. The inhibitory effect of DDP on cell proliferation was high in STEAP4 overexpression HCC cells compared to vehicle group (Fig. 2d). In contrast, the inhibitory effect of DDP on cell proliferation was low in STEAP4 knockdown HCC cells compared to scramble group (Fig. 3c), suggesting STEAP4 promotes the inhibitory effect of DDP on cell proliferation. In addition, we found STEAP4 overexpression significantly reduced the colony number compared to vector control, while STEAP4 knockdown significantly increased colony number compared to scramble control (Figs. 2d and 3c), suggesting STEAP4 inhibits HCC proliferation. Apoptosis assay DDP treatment induced apoptosis. The induction effect of DDP on apoptosis was increased in STEAP4 overexpression cells (Fig. 2e). In contrast, the induction effect of DDP on apoptosis was reduced in STEAP4 knockdown cells (Fig. 3d), suggesting STEAP4 promotes DDPP-induced apoptosis. Meanwhile, we found STEAP4 overexpression increased apoptosis, while STEAP4 knockdown reduced apoptosis, suggesting STEAP4 also promotes apoptosis (Figs. 2e and 3d). These results suggested STEAP4 inhibited chemotherapy resistance.

**Fig. 4:**
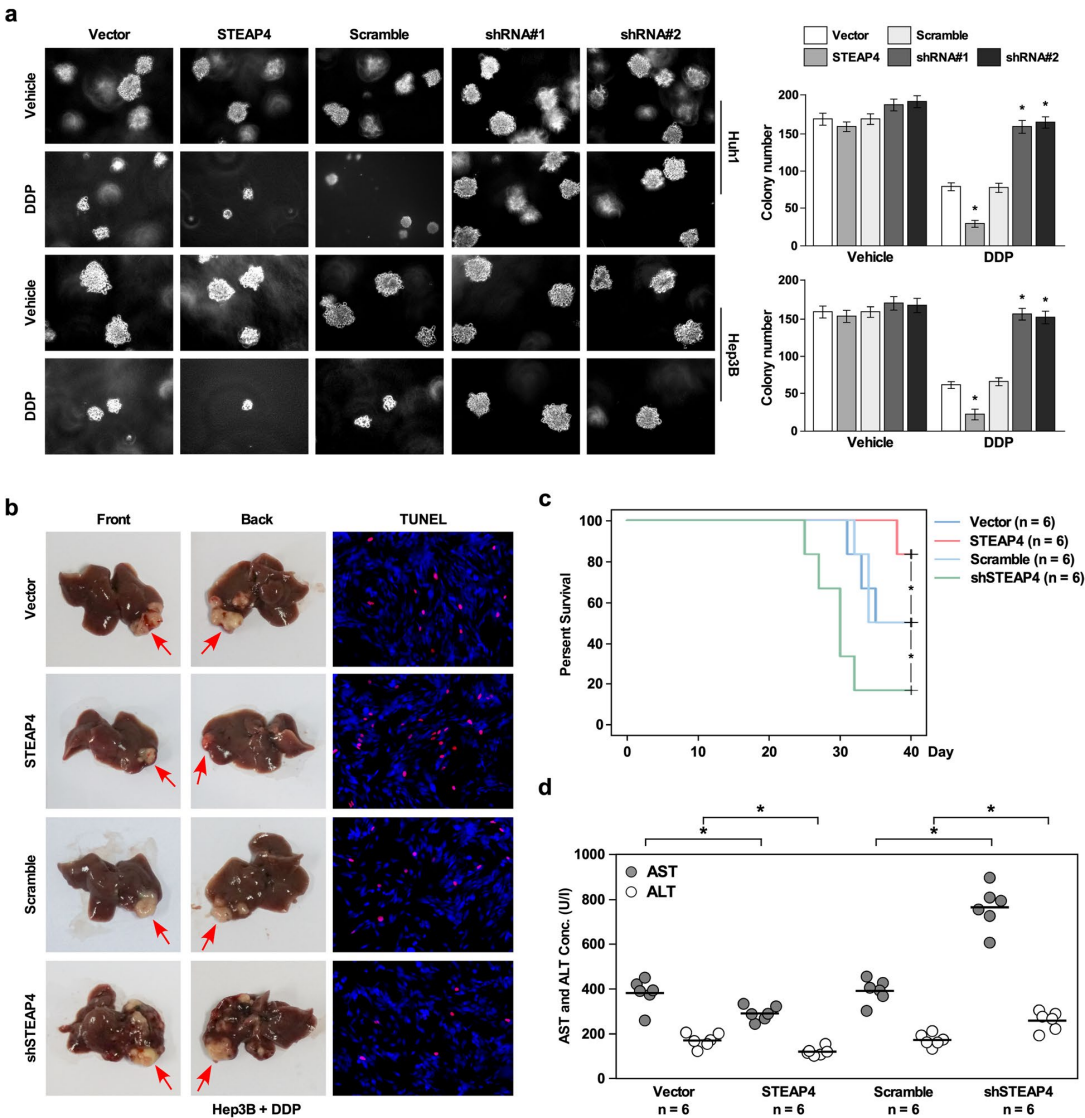
STEPA4 inhibits DDP-induced chemotherapy resistance *in vivo*. **a** Soft agar growth assay for the effect of STEAP4 overexpression or knockdown in DDP-induced cell growth. **b** Representative tumor images of orthotopic transplantation mouse model using STEAP4 overexpression or knockdown HCC cells treated with DDP. TUNEL assay was used to analyze the apoptosis of xenograft tumor tissues. **c** Kaplan-Meier curves with log rank test for orthotopic transplantation mouse model. **d** The concentration of AST and ALT of orthotopic transplantation mouse model. Bars represent the mean ± SD of three independent experiments; *P < 0.05

To confirm above results, we used soft agar growth assay and animal model to analyze the role of STEAP4 on chemotherapy resistance. Soft agar growth assay showed that STEAP4 overexpression inhibited chemotherapy resistance induced by DDP, reflecting in growth inhibition, while STEAP4 knockdown promoted chemotherapy resistance induced by DDP, reflecting in growth promotion (Fig. 4a). Animal model analysis showed STEAP4 overexpression inhibited DDP-induced chemotherapy resistance, reflecting in smaller tumors compared to vector control group. While STEAP4 knockdown promoted DDP-induced chemotherapy resistance, reflecting in larger tumors compared to scramble control group, TUNEL assay also supported this phenotype. STEAP4 overexpression promoted apoptosis induced by DDP, while STEAP4 knockdown inhibited apoptosis induced by DDP (Fig. 4b). In addition, we found STEAP4 inhibited soft agar growth (Fig. 4a), suggesting STEAP4 inhibits cell proliferation. Survival analysis showed the survival time of mice with STEAP4 overexpression was significantly longer than those with STEAP4 knockdown after DDP treatment (Fig. 4c). Aspartate aminotransferase (AST) and alanine aminotransferase (ALT) are markers for liver injury. We found STEAP4 overexpression significantly reduced the concentration of AST and ALT, while STEAP4 knockdown significantly increased the concentration of AST and ALT (Fig. 4d). These findings confirmed STEAP4 inhibited DDP-induced chemotherapy resistance.

### STEAP4 inhibits DDP-induced chemotherapy resistance through inhibiting pi3k/AKT pathway

**Fig. 5:**
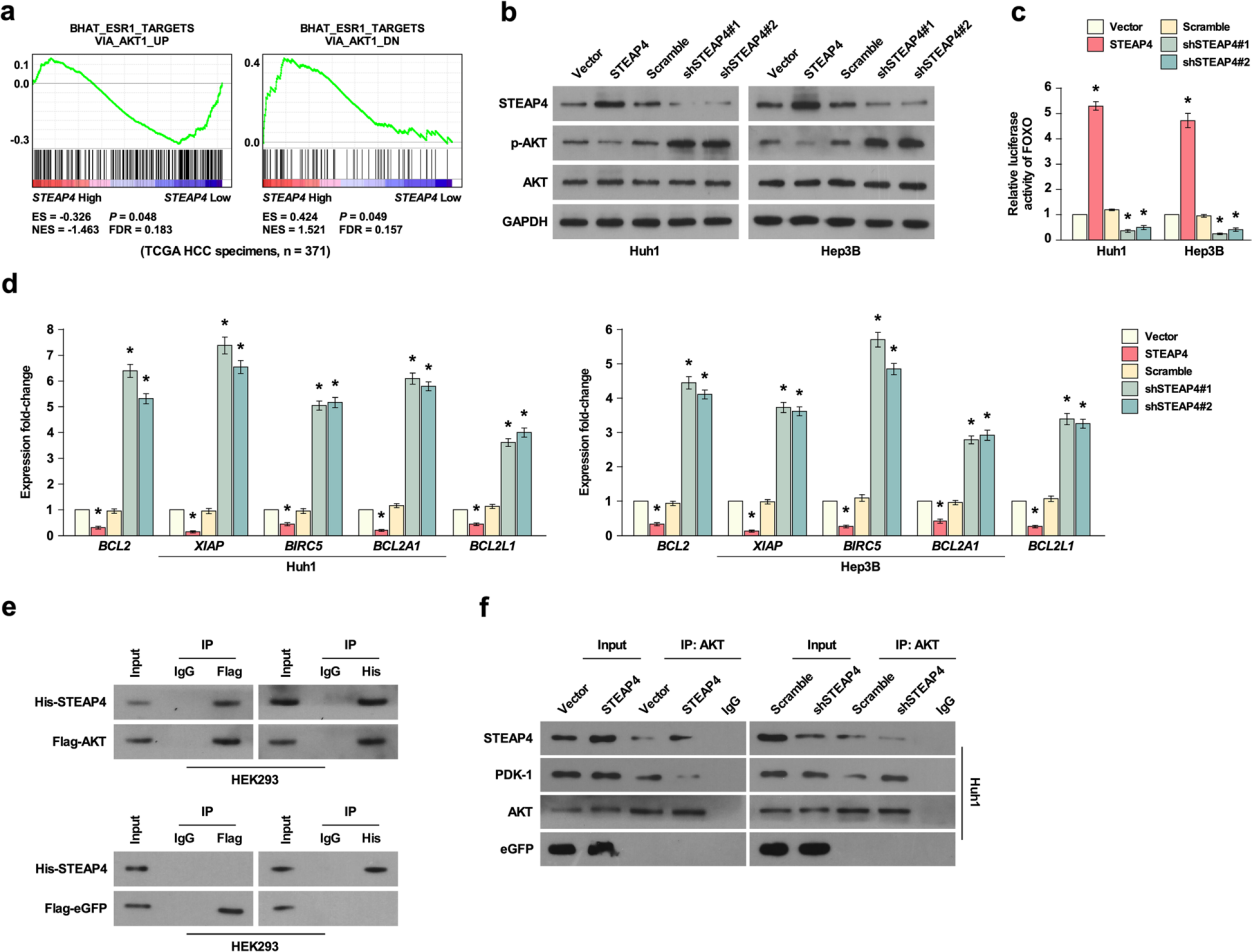
STEAP4 inhibited PI3K/AKT pathway activity. **a** Analysis of the correlation between STEAP4 expression and AKT levels using GSEA. **b** Western blot analysis of AKT and p-AKT expression in cells with STEAP4 overexpression or knockdown. GAPDH was used as the loading control. **c** Luciferase reporter analysis of FOXO transcriptional activity. **d** Q-PCR analysis of BCL2, XIAP, BIRC5, BCL2A1, and BCL2L1 expression in cells with STEAP4 overexpression or knockdown. **e** Co-IP analysis of STEAP4 and AKT interaction in HEK293 cells. **f** Co-IP analysis of STEAP4 and AKT interaction in HCC cells. PDK1 which has been found to interact with AKT was used as the positive control. Bars represent the mean ± SD of three independent experiments; *P < 0.05

To elucidate the regulatory mechanism of STEAP4 inhibiting DDP-induced chemotherapy resistance, we used GSEA to analyze the signaling regulated by STEAP4 and found STEAP4 expression was negatively correlated with the expression of targets of PI3K/AKT1 pathway (Fig. 5a). To confirm this conference, Western blot was used to analyze the phosphorylation of AKT, which is a marker for PI3K/AKT pathway activation [15]. STEAP4 overexpression inhibited the phosphorylation of AKT. STEAP4 knockdown increased the phosphorylation of AKT, while the expression of AKT was not changed, suggesting STEAP4 inhibited PI3K/AKT pathway (Fig. 5b). FOXO transcription factors are major substrates of PI3K/AKT pathway, and they induced cell cycle arrest, stress resistance, and apoptosis [16]. STEAP4 overexpression inhibited the activity of FOXO, while STEAP4 knockdown increased the activity of FOXO, suggesting STEAP4 regulated PI3K/AKT pathway (Fig. 5c). BCL2, XIAP, BIRC5, BCL2A1, and BCL2L1 are the targets of PI3K/AKT pathway. They are associated with apoptosis, and BCL2, XIAP, BIRC5, BCL2A1, and BCL2L1 inhibit apoptosis [17–21]. q-PCR analysis showed that STEAP4 overexpression significantly inhibited their expression, while STEAP4 knockdown significantly increased their expression (Fig. 5d), suggesting STEAP4 induced apoptosis. To further elucidate the molecular mechanism, we determined whether STEAP4 directly interacted with AKT. Co-IP analysis showed that STEAP4 interacted with AKT in 293T (Fig. 5e). To confirm this result, we determined whether AKT interacted with STEAP4 in HCC cells with STEAP4 overexpression or knockdown, and AKT interaction with STEAP4 was reduced in Huh1 with STEAP4 overexpression compared to those expressing empty vector, but the interaction between AKT and PDK1 was reduced. While STEAP4 knockdown reduced the interaction of AKT and STEAP4, but the interaction of PDK1 and PDK1 was increased (Fig. 5f). PDK1 binds to PIP3 at the plasma membrane, which can phosphorylate AKT at Thr^308^, suggesting STEAP4 overexpression reduced the interaction of AKT and PDK1, reducing the phosphorylation of AKT.

**Fig. 6:**
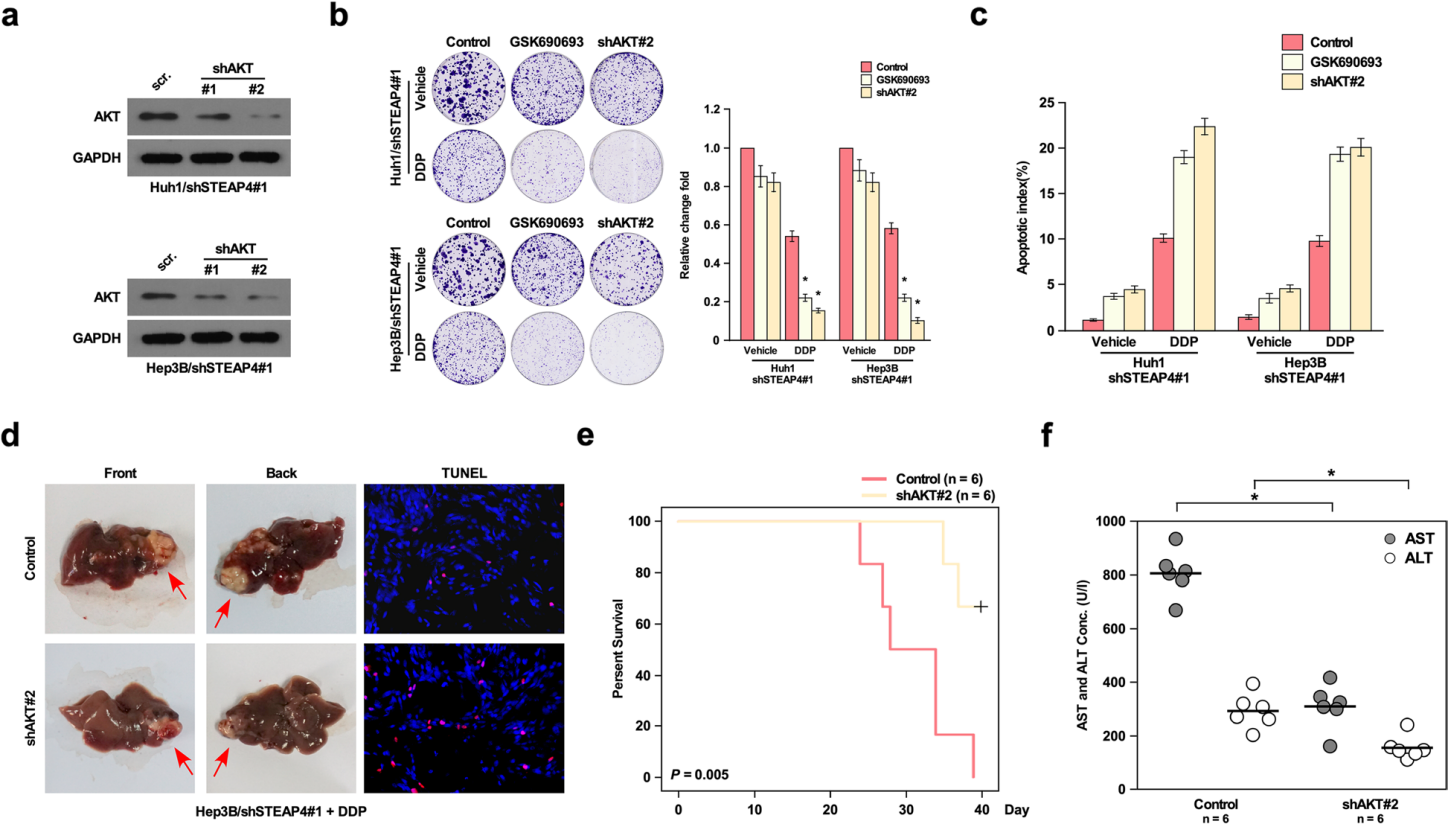
STEAP4 inhibits cisplatin-induced chemotherapy resistance through inhibiting PI3K/AKT pathway. **a** Western blot analysis of AKT expression in AKT and STEAP4 knockdown cells. **b** Colony formation assay of the effect of DDP-induced chemotherapy in AKT and STEPA4 knockdown cells. **c** Apoptosis analysis of the effect of DDP-induced chemotherapy in AKT and STEPA4 knockdown cells. **d** Representative tumor images of orthotopic transplantation mouse model grafted STEAP4 and AKT knockdown cells. TUNEL assay was used to analyze the apoptosis of xenograft tumor tissues. **e** Kaplan-Meier curves with log rank test for orthotopic transplantation mouse model grafted with cells with STEPA4 and AKT knockdown. **f** The concentration of AST and ALT of orthotopic transplantation mouse model grafted with cells with STEPA4 and AKT knockdown. Bars represent the mean ± SD of three independent experiments; *P < 0.05

To determine whether STEAP4 inhibited chemotherapy resistance through inhibiting PI3K/AKT pathway, we knocked down AKT in STEAP4 knockdown cells (Fig. 6a), colony formation assay showed that inhibition of AKT and STEAP4 significantly increased DDP-induced proliferation arrest (Fig. 6b), and apoptosis assay showed that inhibition of AKT and STEAP4 also increased DDP-induced apoptosis (Fig. 6c). Animal model *in vivo* showed that DDP inhibited the growth of tumors derived from Hep3B with double inhibition of AKT and STEAP4 compared to tumors derived from Hep3B with only inhibition of STEAP4. TUNEL assay also showed inhibition of AKT, and STEAP4 also promoted DDP-induced apoptosis in tumor tissues (Fig. 6d). Kaplan-Meier analysis mice with double inhibition of AKT and STEAP4 had longer survival time than mice with only STEAP4 knockdown (Fig. 6e). The concentration of AST and ALT was also reduced in mice with double inhibition of AKT and STEAP4 compared to mice with only inhibition of AKT (Fig. 6f). These findings suggested STEPA4 inhibited DDP-induced chemotherapy resistance through inhibiting AKT activation.

**Fig. 7:**
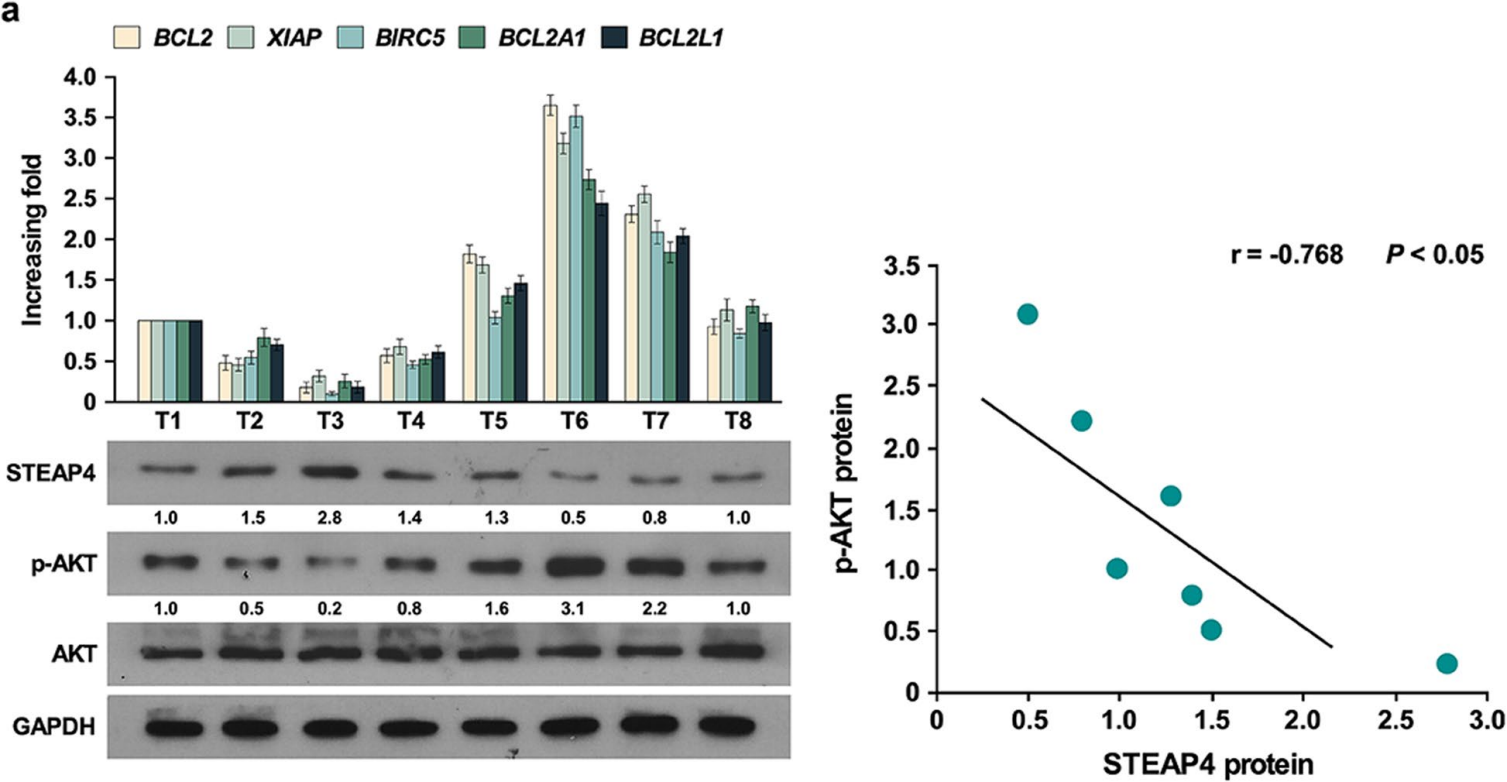
STEAP4 levels were negatively correlated with PI3K/AKT pathway activity in clinic specimens. **a** Q-PCR analysis of BCL2, XIAP, BIRC5, BCL2A1, and BCL2L1 expression in HCC tissues, Western blot analysis of STEAP4, AKT, and p-AKT expression in HCC tissues. GAPDH was used as the loading control. Statistical analysis of the correlation of p-AKT expression and STEAP4 expression. Bars represent the mean ± SD of three independent experiments

To further confirm above results, we determined the relationship between STEAP4 and AKT activation in clinical samples. q-PCR was used to determine the expression of AKT pathway target genes in eight HCC tissues, such as BCL2, XIAP, BIRC5, BCL2A1, and BCL2L1. Western blot was used to determine the expression of AKT and phosphorylation of AKT in clinical samples. STEAP4 expression negatively correlated with phosphorylation of AKT (Fig. 7), which further strengthened the finding that STEAP4 inhibited DDP1-induced chemotherapy resistance through inhibiting AKT activity.

## Discussion

The chemotherapy resistance is the biggest obstacle for HCC therapy. The first-line drugs were often been reported to generate chemotherapy resistance, such as sorafenib [22] and platinum-based drugs [23]. So, exploring the regulatory mechanism of chemotherapy resistance generation is the urgent need for HCC therapy.

Through analyzing TCGA dataset of HCC specimens and clinic information, we found STEAP4 level was reduced in patients with recurrence, suggesting STEAP4 might inhibit chemotherapy resistance, so we started to determine whether STEAP4 regulated cisplatin-induced chemotherapy resistance. In present study, we found STEAP4 was downregulated in HCC cells and tissues. Patients with low STEAP4 had poor prognosis. Functional analysis suggested STEAP4 promoted cisplatin-induced growth arrest and apoptosis. Mechanism analysis suggested STEAP4 inhibited the phosphorylation of AKT, and the expression of target genes of PI3K/AKT pathway was also reduced, suggesting STEAP4 inhibited PI3K/AKT pathway. Then, we determined whether AKT directly interacted with STEAP4 and found STEAP4 directly interacted with AKT, suggesting STEAP4 inhibited PI3K/AKT pathway through directly interacting with AKT. To confirm whether STEAP4 inhibited cisplatin-induced chemotherapy through inhibiting PI3K/AKT pathway, we doubly knocked down AKT and STEAP4 in HCC cells, function analysis showed double knockdown of AKT, and STEAP4 promoted cisplatin-induced chemotherapy, suggesting STEAP4 inhibited cisplatin-induced chemotherapy through inhibiting PI3K/AKT pathway. We also analyzed the relationship between STEAP4 expression and PI3K/AKT pathway activity in clinic specimens and found STEAP4 expression was negatively correlated with AKT activity in specimens. Our findings suggested STEAP4 could function as tumor suppressor, providing a new target for HCC therapy.

Many studies show PI3K/AKT pathway regulates chemotherapeutic drug resistance in HCC [24, 25]; our study also suggested STEAP4 inhibited HCC chemotherapy resistance through suppressing PI3K/AKT pathway.

## Conclusions

In summary, we found STEAP4 was low in HCC cells and tissues, patients with low STEAP4 had poor prognosis, and it is an independent prognostic factor for HCC patients and inhibited HCC chemotherapy resistance through suppressing PI3K/AKT pathway.

## Data Availability

All data generated or analyzed during this study are included either in this article.

## Abbreviations

HCC: Hepatocelluar carcinoma
RT-qPCR: Reverse transcription quantitative polymerase chain reaction
IHC: Immunohistochemistry
ATCC: American Type Culture Collection
TUNEL: Terminal deoxynucleotidyl transferase nick end labeling
shRNA: Short hairpin RNA
