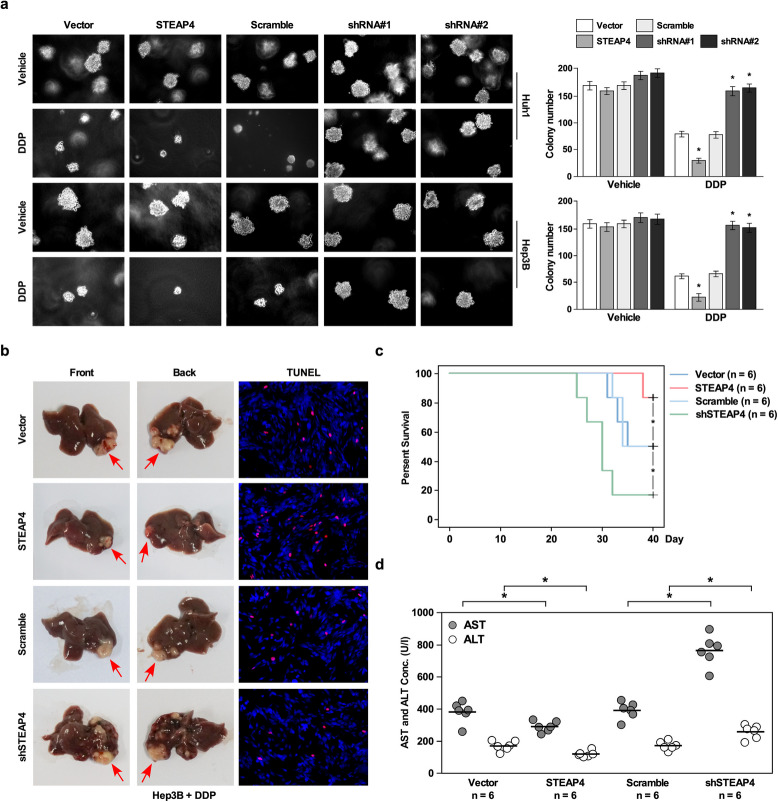# Correction: STEAP4 inhibits cisplatin-induced chemotherapy resistance through suppressing PI3K/AKT in hepatocellular carcinoma

**DOI:** 10.1186/s40170-024-00356-0

**Published:** 2025-03-10

**Authors:** Binhui Xie, Baiyin Zhong, Zhenxian Zhao, Jie Hu, Jianqiong Yang, Yuankang Xie, Jianhong Zhang, Jianting Long, Xuewei Yang, Heping Li

**Affiliations:** 1 Department of Hepatobiliary Surgery, the First Affiliated Hospital of Gannan Medical University, Ganzhou, 341000 People’s Republic of China; 2 Ganzhou Key Laboratory of Hepatocellular Carcinoma, the First Affiliated Hospital of Gannan Medical University, Ganzhou, 341000 People’s Republic of China; 3 Department of Hepatobiliary Surgery, The First Affiliated Hospital of Sun Yat-Sen University, Guangzhou, 510080 People’s Republic of China; 4 Department of Medical Oncology, The First Affiliated Hospital of Sun Yat-Sen University, Guangzhou, 510080 People’s Republic of China; 5 Department of Clinical Research Center, the First Affiliated Hospital of Gannan Medical University, Ganzhou, 341000 People’s Republic of China; 6 Department of Hepatobiliary Surgery, the Second Affiliated Hospital of Guangzhou Medical University, Guangzhou, 510000 People’s Republic of China

**Correction** **:** **Cancer Metab 11, 26 (2023)**

**https://doi.org/10.1186/s40170-023-00323-1**

Due to an error in figure assembly the original [1] publication of this article contained an incorrect figure 4a. The incorrect and correct figure are shown in this correction article. The original article has been updated.

Incorrect figure 4


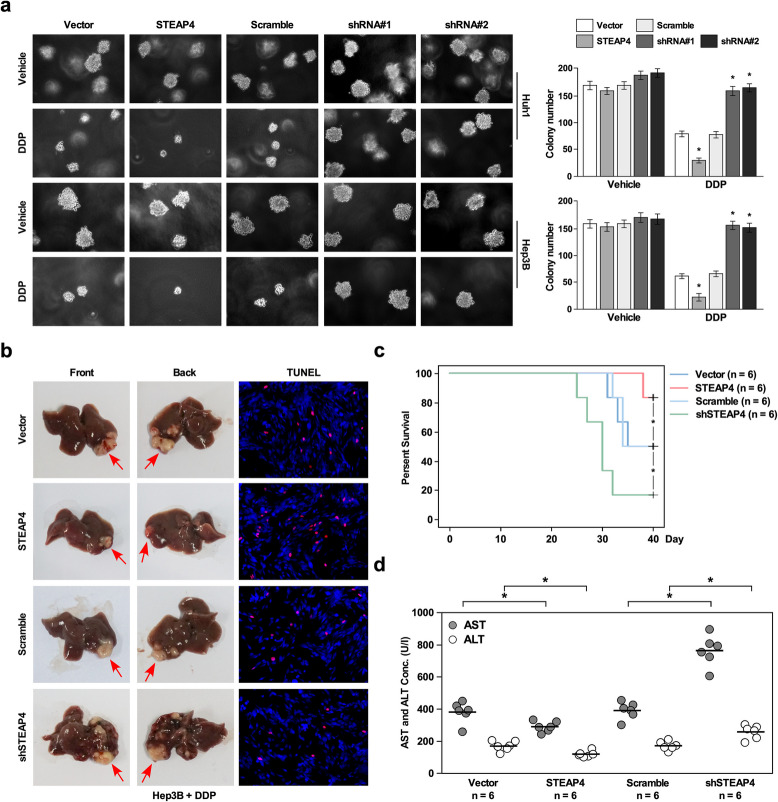


Correct figure 4